# Improvement of Gelatin-Based Coating in Order to Enhance the Oxygen Barrier and Mechanical Properties of Biobased PLA (Polylactic Acid) and Cellulose Films

**DOI:** 10.1021/acsomega.5c00198

**Published:** 2025-07-14

**Authors:** Mine Kulalı Gül, Hayati Türkmen

**Affiliations:** a Department of Material Science and Engineering, 37509 Ege University, Bornova Izmir 35100, Turkey; b Bak Ambalaj, 10002. Sk. No. 45, Atatürk Osb, Çiğli, İzmir 35620, Turkey; c Department of Chemistry, Faculty of Science, 37509 Ege University, Bornova, Izmir 35100, Turkey

## Abstract

This study presents a gelatin-based coating designed to improve the barrier and mechanical properties of biobased films such as polylactic acid (PLA) and cellulose. A formulation consisting of 8 wt % gelatin, 2 wt % glycerol, and 0.1 wt % Tween 80 was found to exhibit favorable barrier and mechanical properties based on parametric studies. Coated compostable films were evaluated under controlled conditions (80% RH, 23 °C). Oxygen permeability (OTR) was significantly reduced, from >200 to 16.19 cm^3^/m^2^/day for PLA and from 2.20 to 0.64 cm^3^/m^2^/day for cellulose, indicating an over 90% improvement. Tensile tests confirmed increased durability and load-carrying capacity, while the coating enhanced surface scratch resistance without compromising optical clarity. Spectral and color analyses showed that the Δ*E* values ranged from 2.66 to 2.73, remaining below the Δ*E* < 5 threshold, thereby confirming their suitability for printing applications. These findings demonstrate that gelatin-coated biobased films offer remarkable oxygen barrier properties, enhanced mechanical strength, and consistent optical characteristics, making them viable for sustainable packaging. By improving biodegradable film performance without sacrificing printability, this study contributes to reducing the environmental footprint of flexible packaging and advancing a circular economy.

## Graphical abstract

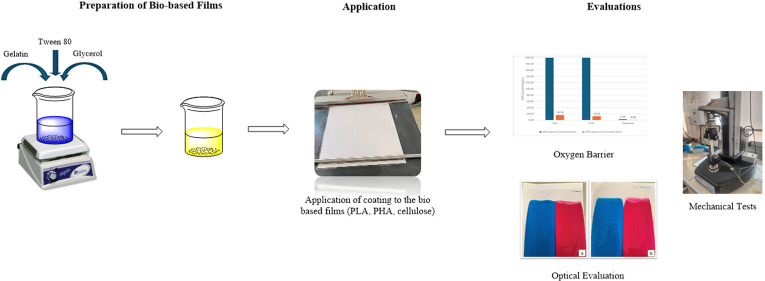

## 1 Introduction

Environmental pollution and sustainability remain major global challenges. The depletion of natural resources, increasing waste accumulation, and environmental degradation threaten ecosystems and future generations. The rapid expansion of flexible packaging in the food industry has significantly contributed to plastic waste, which is difficult to manage and persists in nature, impacting terrestrial and marine ecosystems.^1^ In 2022, global plastic production reached 390.7 million tons, and if current trends continue, approximately 12,000 MT of plastic waste could accumulate in nature by 2050.^2^ Packaging alone accounts for nearly 40% of total plastic waste,^3^ emphasizing the urgent need for sustainable alternatives.

Plastics, primarily derived from petroleum, are widely used in food packaging due to their cost effectiveness, durability, and strong barrier properties. However, conventional flexible packaging materials such as PET (polyethylene terephthalate), PE (polyethylene), and PP (polypropylene) pose long-term environmental risks due to their low degradability.^4,5^ While recycling efforts aim to mitigate plastic waste, the complexity of multilayered flexible packaging structures limits their recyclability.^6^ Factors such as inadequate infrastructure, economic constraints, and inefficient waste collection further hinder large-scale recycling adoption.^7^

To reduce plastic pollution, research has focused on biodegradable and compostable materials such as polylactic acid (PLA) and cellulose. These materials provide more environmentally friendly options; however, their mechanical strength and oxygen barrier properties are generally inferior to those of petroleum-based plastics.^8^ Enhancing these properties is crucial for their broader application in the packaging industry.

This study explores a gelatin-based biocoating designed to improve the mechanical and oxygen barrier properties of PLA and cellulose films. Gelatin was selected due to its biodegradability, low oxygen permeability, and emulsification properties.^9,10^ The coating’s effectiveness was evaluated through mechanical, optical, and oxygen permeability tests, with results compared against commercial films. By enhancing the performance of biobased films, this study aims to contribute to the development of sustainable flexible packaging solutions that align with circular economy principles and help reduce the environmental impact of plastic packaging.

## 2 Materials and Methods

### 2.1 Coating Formulation

The coating formulation was prepared as a gelatin-based system. Bovine gelatin (CAS number 9000-70-8) was used in this study due to its high gel-forming ability, biodegradability, and low manufacturing cost.^11^ It was supplied by SelJel Jelatin A.Ş. Glycerol, supplied by Sigma-Aldrich, was used as a plasticizer. Tween 80 (Polysorbate 80), purchased from Merck, was used as an emulsifier to ensure the homogeneous distribution of the formulation and facilitate its spreading. Distilled water was used as the solvent in the formulations.

First, the gelatin was weighed in the predetermined amount and added to a conical flask. Then, 50 mL of distilled water was added. The mixture was stirred with a magnetic stirrer at 50 °C for 30 min at 500 rpm. After the gelatin dissolved in water, glycerol, and Tween 80 were added, and the solution was mixed for another 30 min at 50 °C and 500 rpm.

Three different sets of experiments were conducted to identify a suitable coating formulation, with varying amounts of gelatin, glycerol, and Tween 80 (Table 1). To evaluate the gelatin ratio in the coating formulation, formulations with increasing gelatin ratios of 3, 4, 5, 6, 8, and 10% by weight were prepared (entries 1–10). The coatings were applied to a biobased film to examine the effect of the gelatin ratio on the film’s oxygen barrier properties and to identify suitable gelatin concentration. In the second step, different glycerol ratios were tested using the selected gelatin ratio of 8%. The impact of these glycerol variations on the film’s oxygen barrier properties was evaluated to determine a composition yielding favorable performance. Coating formulations were prepared by testing glycerol amounts of 1, 2, and 4%, alongside the 1% glycerol ratio, under experimental set 2.

**Table 1:** Sets of Experiments for the Coating Formulations

entry	gelatin (%)	glycerol (%)	Tween 80 (%)
1	3	1	0.1
2	4	1	0.1
3	5	1	0.1
4	6	1	0.1
5	8	1	0.1
6	10	1	0.1
7	8	2	0.1
8	8	4	0.1
9	8	1	0.5
10	8	1	1

In the third experimental set, the amount of Tween 80 was evaluated. The concentration of Tween 80 is critical for achieving the desired properties of the coating. To assess the amount of Tween 80 required for homogeneous distribution of the coating, ratios of 0.1, 0.5, and 1% were tested.

### 2.2 Preparation of Coated Films

Different compostable films were used to apply the coating formulations. Specifically, PLA (polylactic acid) and cellulose films were selected. The cellulose film used is a modified form that incorporates polyvinylidene chloride (PVdC). The use of PVdC-modified cellulose film provides an inherent oxygen barrier, and by applying a gelatin coating, this study aims to investigate how the additional layer further enhances oxygen barrier performance, offering insights into the improvements achievable through this biobased coating.

The K Control Coater System K 202 device was utilized to apply the prepared coating formulations onto the films. Figure 1 illustrates the stages involved in applying the coating using the control coater.

**Figure 1:**
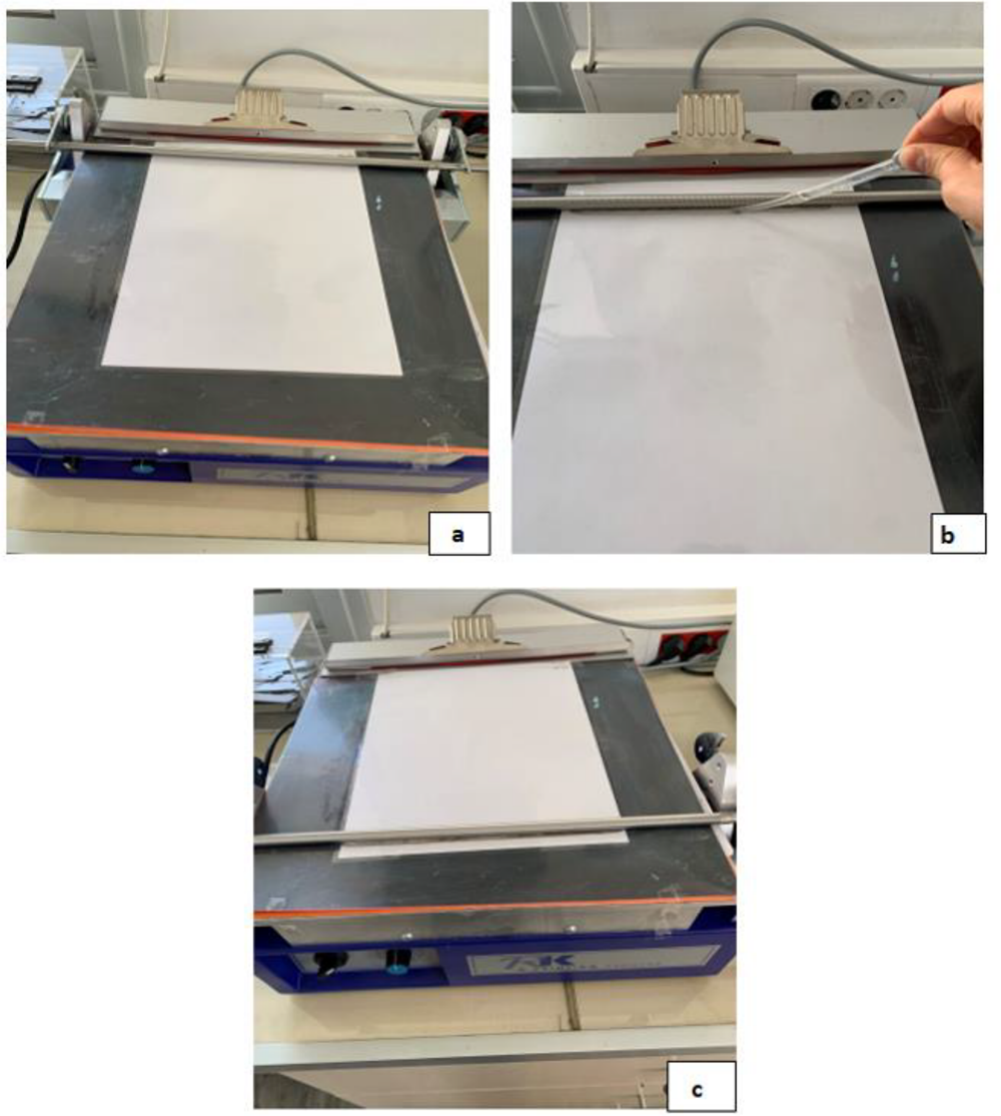
(a) Placement of the sample into the film coater device. (b) Application of lacquer onto the film surface in front of the K-bar using a pipet. (c) Coating of the film surface by moving the K-bar.

First, the film to be coated is placed onto the base of the control coater device. To prevent slipping, a sheet of paper is placed underneath the film and secured with tape (Figure 1a). Next, a spiral wire-wound rod, known as a K-bar, is positioned and fixed in place using corner clips. The prepared coating solution is then dispensed in front of the K-bar using a pipet (Figure 1b). With the machine speed set to 3 rpm, the device is activated, and the K-bar begins to rotate and move across the film surface. The spiral wire-wound structure of the K-bar picks up the coating solution and spreads it evenly across the film. In this way, the film becomes uniformly coated (Figure 1c). After coating, the films are placed in an oven at 50 °C for 30 min to cure the coating.

### 2.3 Characterization

After all the coated films were prepared, the oxygen transmission rate (OTR) was measured using the MOCON OX-TRAN 2/22H at 23 °C and 80% relative humidity (RH) to evaluate oxygen permeability, following ASTM D3985 standards. A 50 cm^2^ sample was cut and fixed between two plates in the device. The plates with the sample were placed in the chamber, and the measurements were repeated twice to ensure accuracy. A lower oxygen transmission rate (OTR) indicates better barrier properties of the packaging. Therefore, a lower OTR value is more desirable.

A Lloyd LS1 material testing machine was utilized to perform tensile strength and puncture tests, in accordance with ASTM D882 for tensile strength and ASTM F1306 for puncture resistance. For tensile strength testing, 25 mm-wide samples were placed between the two jaws of the device. The test was conducted by applying a uniaxial tensile force, recording the stress and extension behavior until the sample fractured. For puncture resistance measurements, a separate clamping apparatus was used with the tensile testing device. Samples (16 cm × 16 cm) were clamped securely in the fixture, and force was applied using a piercing needle until the film was punctured. The maximum applied force was recorded as the puncture strength. Mechanical and color measurements were repeated three times to ensure data reliability. Color measurements were conducted with an X-Rite eXact spectrophotometer to analyze *L*, *a*, and *b* values. A BYK haze-gard plus device was used for haze measurements. The haze level of a material is determined by measuring the light scattered by particles on or within its surface. Transparent films can appear hazy or clear depending on their light scattering behavior. Therefore, haze measurements are conducted alongside clarity and transmittance evaluations, providing additional data on these optical properties.^6^ All measurements were conducted using three samples per test to obtain representative average values. Standard deviations were also calculated from the repeated measurements to assess the variability and reliability of the data.

## 3 Results and Discussion

### 3.1 Oxygen Barrier Evaluation

#### 3.1.1 Effect of Coating Unit Weight to the Oxygen Barrier

To observe the effect of application weight on the barrier properties before considering the polymer ratio, the coating formulation mixture was applied to the film using K-bars with different screen widths numbered 2, 3, and 4, while keeping the gelatin ratio constant. In the coater application using the formulation prepared with a 4% gelatin ratio, the coating was applied with the K-Bar type, and the weight of the coating was measured after drying, based on the difference between the coated and uncoated films. The weights of the dried coatings were measured as follows: K-Bar 2 had a weight of 1.04 g, K-Bar 3 had a weight of 1.35 g, and K-Bar 4 had a weight of 1.62 g. The differences in weights among the K-Bars are attributed to their varying screen depths, which result in different application weights. This variation is also influenced by the water content in the formulation, with only the dry matter remaining on the film after drying. In the coating application containing 4% gelatin, the lowest OTR value measured was 84.36 cm^3^/m^2^/day with K-Bar 4. It was observed that as the dry weight of the coating on the film increased, the OTR value decreased, indicating an improvement in the oxygen barrier of the film. Therefore, four K-Bars were used in the coater applications to assess the effect of coating formulations with different gelatin ratios on the oxygen barrier.

#### 3.1.2 Effect of Amount of Gelation on the Oxygen Barrier

To observe the effect of different gelatin ratios on the oxygen barrier, entries 1–7 were prepared and applied to PLA film using a K-Bar 4. Two replicate OTR tests were conducted on the coated films. Figure 2 illustrates the changes in average OTR values according to the gelatin ratio.

**Figure 2:**
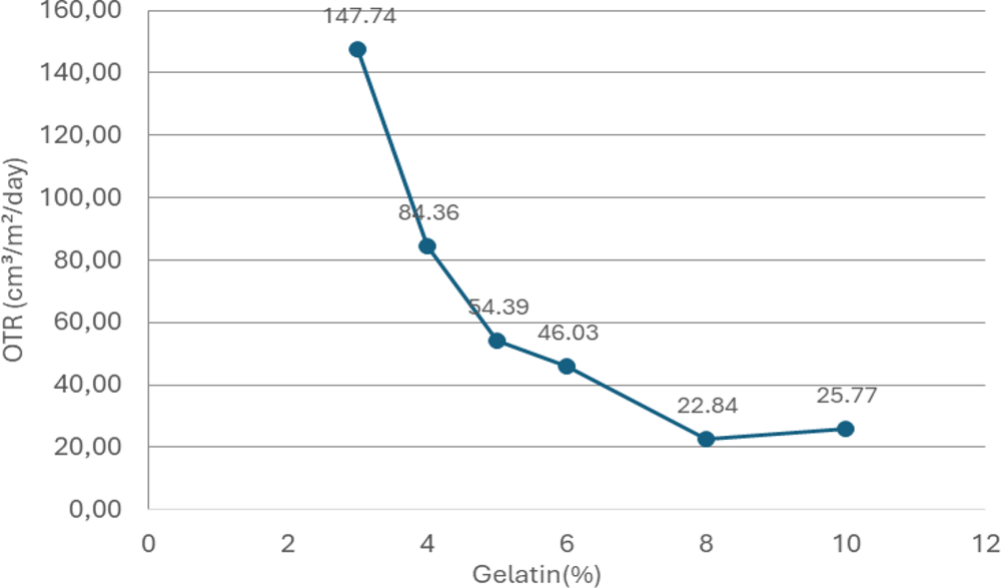
OTR values of coated PLA films according to different gelatin ratios.

The curve in Figure 2 shows that as the gelatin ratio increases, the OTR value decreases, indicating a corresponding reduction in oxygen permeability, as expected. This trend is consistent with the findings of Li et al.^12^ and Debeaufort et al.,^13^ who demonstrated that gelatin coatings effectively enhance oxygen barrier properties. However, while the oxygen permeability of the film coated with 8% gelatin (entry 5) was measured at 22.84 cm^3^/m^2^/day, the OTR value for the 10% gelatin-coated film (entry 6) was higher, at 25.77 cm^3^/m^2^/day. Typically, increasing the gelatin concentration should improve the oxygen barrier, but the higher permeability observed at 10% gelatin may be due to decreased homogenization in the coating formulation or challenges in applying the coating solution uniformly, as noted by Debeaufort et al.^13^

For each formulation, two independent OTR measurements were conducted, and the variation between replicates was evaluated. The low variation confirms the consistency of the experimental replicates. The calculated OTR values are as follows: entry 1 (3% gelatin), 147.74 ± 38.81 cm^3^/m^2^/day; entry 2 (4% gelatin), 84.36 ± 8.05 cm^3^/m^2^/day; entry 3 (5% gelatin), 54.39 ± 9.59 cm^3^/m^2^/day; entry 4 (6% gelatin), 46.03 ± 2.43 cm^3^/m^2^/day; entry 5 (8% gelatin), 22.84 ± 0.69 cm^3^/m^2^/day; and entry 6 (10% gelatin), 25.77 ± 8.58 cm^3^/m^2^/day. The lowest standard deviation was observed in entry 5 (8% gelatin), indicating the highest measurement reliability for this formulation.

The relatively low standard deviations across the formulations demonstrate the reproducibility of the results, suggesting that measurement uncertainty did not significantly affect the overall trend. Given the consistency of the results, the average values were used for trend analysis. Overall, these findings confirm that gelatin coatings can significantly improve oxygen barrier properties. However, careful control of gelatin concentration is essential to ensuring the desired effect without compromising coating uniformity. This trend is clearly illustrated in the surface plot graphs in Figure 4.

#### 3.1.3 Effect of Glycerol to the Oxygen Barrier

Initially, 1% glycerol was used in the coating formulation while evaluating the polymer ratios. To examine the effect of glycerol content, formulations with 2 and 4% glycerol were also tested. Following these trials, an OTR test was conducted after applying the coating to the film. Figure 3 presents the OTR test results for the different glycerol ratios.

**Figure 3:**
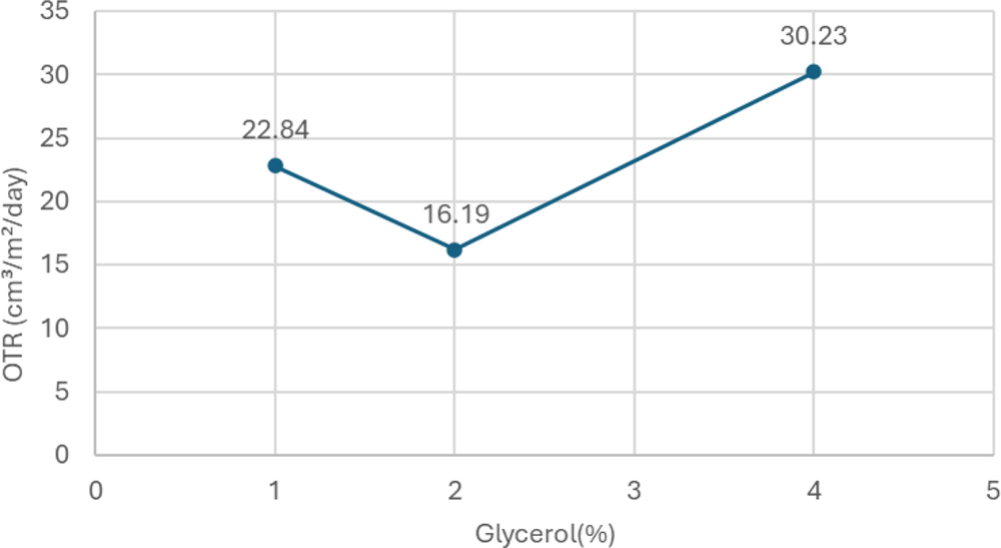
OTR values of coated films with different glycerol ratios in the coating formulation.

Glycerol was incorporated as a plasticizer in the coating formulation to enhance flexibility, softness, and durability. Consistent with previous studies,^14,15^ our results indicate that adjusting glycerol content is important to balancing mechanical and barrier properties. As illustrated in Figure 3, increasing the glycerol concentration from 1 to 2% reduced the oxygen transmission rate OTR from 22.84 ± 0.69 to 16.19 ± 0.68 cm^3^/m^2^/day, suggesting enhanced barrier performance. However, when the glycerol level was further increased to 4%, the OTR rose to 30.23 ± 7.72 cm^3^/m^2^/day, indicating that excessive glycerol compromises the barrier. This trend aligns with previous findings, which highlight that while glycerol enhances flexibility, excessive amounts disrupt the biopolymer matrix, reducing its barrier effectiveness.^14^

When the Tween 80 ratio is kept constant, a surface plot was generated in the Minitab program to observe the effect of glycerol and gelatin amounts on the OTR value (Figure 4a). The graph visually indicates that the OTR value decreases as the amount of gelatin increases. In this study, the results confirm that the lowest OTR value (16.19 ± 0.68 cm^3^/m^2^/day) was achieved at entry 7 (8% gelatin, 2% glycerol, 0.1% Tween 80), suggesting that this formulation offers a favorable combination of flexibility and oxygen barrier performance. Increasing glycerol concentration beyond this level (4%) led to a significant increase in OTR (30.23 ± 7.72 cm^3^/m^2^/day), likely due to structural changes in the film matrix that facilitated oxygen permeability. Therefore, based on these findings, 2% glycerol was identified as the most effective concentration for achieving the lowest OTR while maintaining structural integrity. These results align with recent studies, reinforcing the established role of glycerol plasticization in the performance of biopolymer-based coatings.

**Figure 4:**
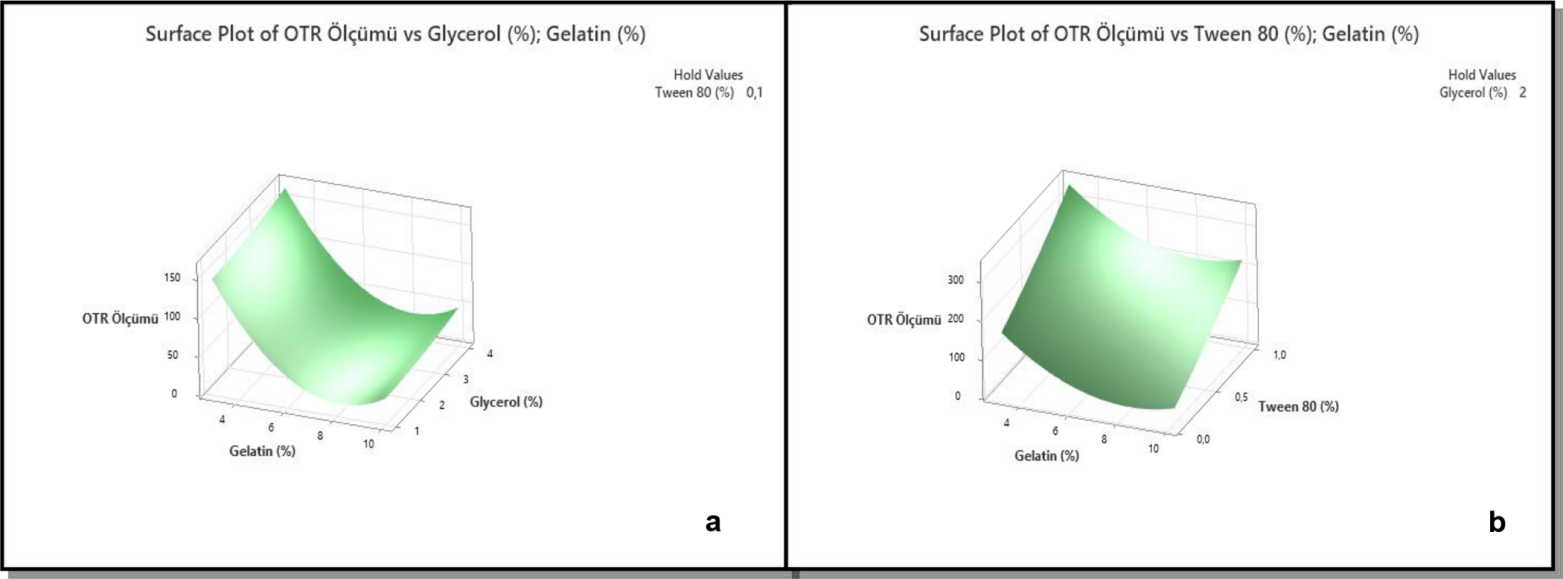
Surface plot graphs of gelatin and glycerol (a); gelatin and Tween 80 (b).

#### 3.1.4 Effect of Tween 80 on the Oxygen Barrier Performance

A concentration of 0.1% Tween 80 was used in the coating formulation where different polymer ratios were tested. To evaluate the effect of Tween 80 on the coating formulation, 8% gelatin was prepared with increased Tween 80 concentrations of 0.5 and 1%. The prepared coatings were applied to the film, and OTR test measurements were taken. Tween 80 facilitates emulsion formation by creating a stable bond between oil and water, which helps achieve a homogeneous structure in the coating. By providing a uniform structure, Tween 80 enhances the surface properties of the coating formulation. However, excessive use of Tween 80 can destabilize the formulation and reduce its durability.^16^

As shown in Figure 4b, while the OTR value was 22.84 cm^3^/m^2^/day (entry 5) with a Tween 80 concentration of 0.1%, increasing the Tween 80 concentration to 0.5% raised the OTR value to 87.5 cm^3^/m^2^/day (entry 9). When the Tween 80 concentration was increased to 1%, the OTR value could not be measured due to an error. Since our OTR device can measure a maximum value of 200 cm^3^/m^2^/day (entry 10), it is likely that the OTR value exceeded 200 cm^3^/m^2^/day at 1% Tween 80. Our experiments showed that 0.1% Tween 80 produced the most favorable barrier performance. Increasing the Tween 80 concentration beyond this point negatively affected the stability of the formulation and reduced the coating’s barrier properties.

#### 3.1.5 Effect of Coating on the Oxygen Barrier Performance of Different Biobased Films

Following the application of gelatin-based coating formulations to PLA film and the measurement of its oxygen barrier properties, the selected formulation was also applied to cellulose films. Barrier values for both coated and uncoated films were measured and compared. Each measurement was repeated twice, and the results are presented in Figure 5.

**Figure 5:**
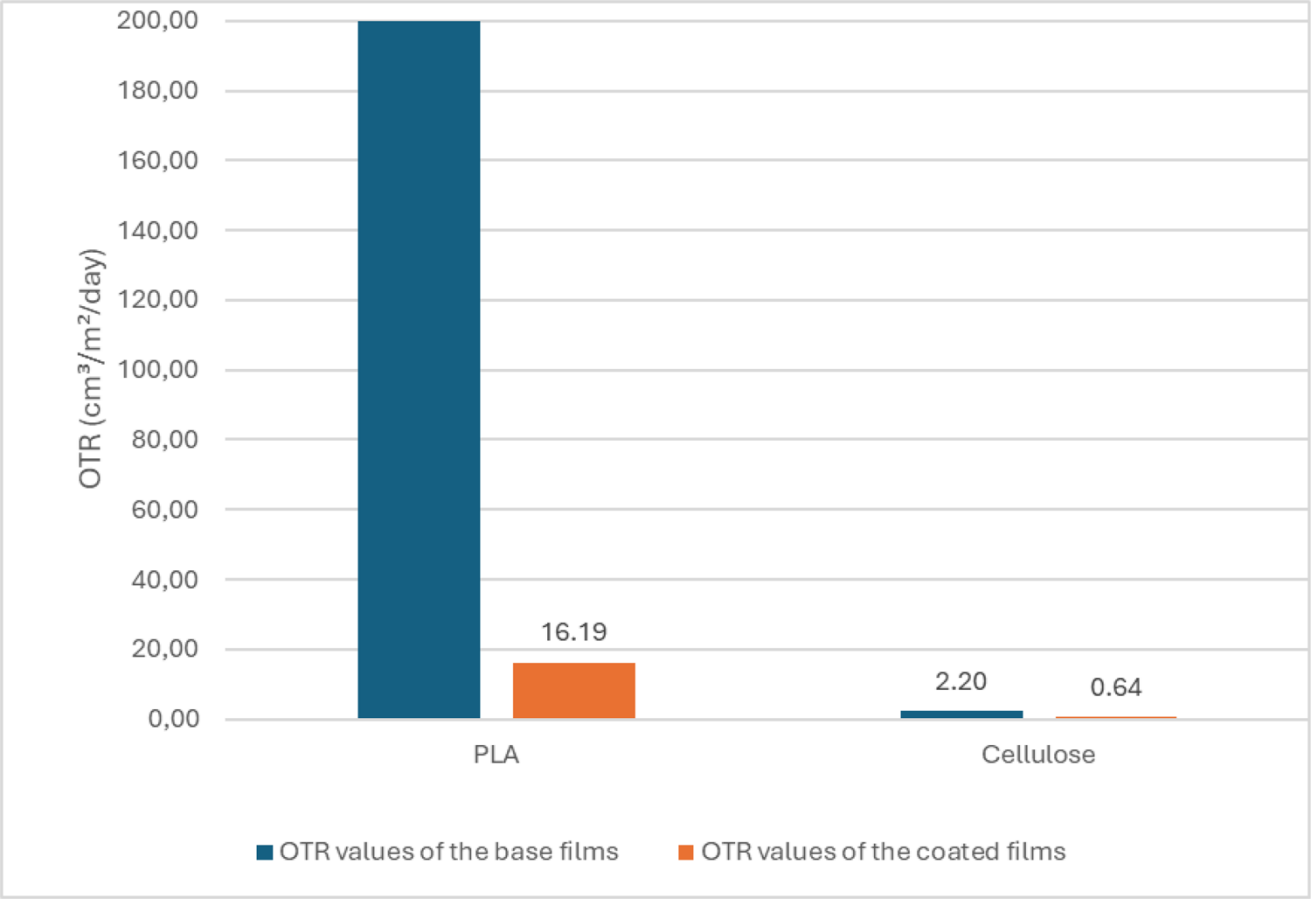
OTR values of coated and uncoated PLA and cellulose films.

The OX-TRAN brand 2/22H model device, which measures oxygen barrier properties, has an operating range of 0.05 to 200 cm^3^/m^2^/day. However, this upper limit of 200 cm^3^/m^2^/day is inadequate for food packaging purposes. Consequently, when assessing the barrier performance of PLA film, the oxygen barrier measurement device encounters inaccuracies, as it has been observed that the oxygen barrier value of uncoated PLA film exceeds 200 cm^3^/m^2^/day. Nevertheless, the barrier efficacy of gelatin-coated PLA film has been reduced to 16.19 cm^3^/m^2^/day, while that of gelatin-coated cellulose film has decreased from 2.20 to 0.64 cm^3^/m^2^/day, as illustrated in Figure 5.

The uncoated film referred to as “cellulose” in this study incorporates PVdC for barrier functionality. To assess the effect of applying a coating to a substrate that already exhibits oxygen barrier properties, its OTR value was measured as 2.20 cm^3^/m^2^/day. After applying the gelatin coating, this value decreased to 0.64 cm^3^/m^2^/day. This substantial reduction in oxygen permeability indicates that the gelatin coating further enhances the barrier performance of already high-barrier films.

The discrepancy in oxygen permeability between the coated PLA and cellulose films may also arise from differences in their chemical compositions. The chemical structure of both the coating and the film substrate influences interfacial interactions, which can enhance adhesion and improve barrier performance. Additionally, surface properties such as roughness, hydrophobicity, and surface energy significantly affect the effectiveness of the coating. These factors may lead to variations in coating uniformity and interaction, ultimately resulting in differences in oxygen permeability.^17^

To compare the oxygen barriers of biobased gelatin-coated and uncoated films with petroleum-based flexible packaging films, which are widely used in the industry, oxygen barriers were tested for 100 μm blown PE, 20 μm BOPP (biaxially oriented polypropylene) film, 12 μm PET (polyethylene terephthalate), 8 μm aluminum, and 20 μm OPA (oriented polyamide) film. The oxygen barrier permeabilities of PET, PE, BOPP, OPA, aluminum films, and biobased gelatin-coated and uncoated films, are shown in Figure 6. All OTR measurements were performed at 23 °C and 80% RH under standardized conditions.

**Figure 6:**
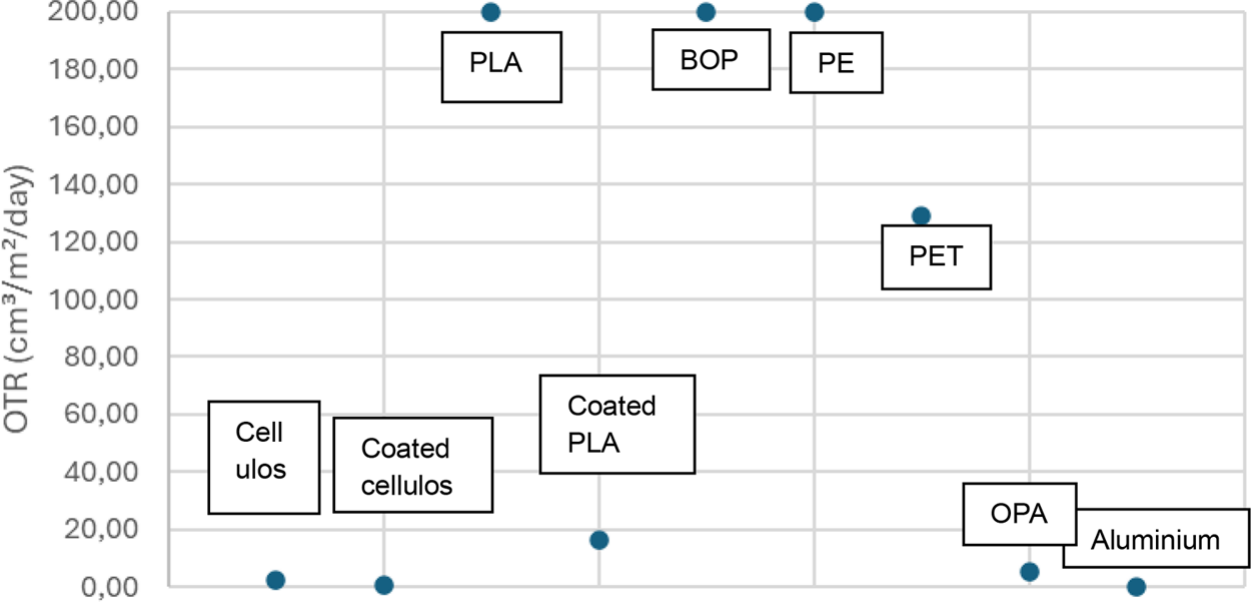
Oxygen permeability of biobased and plastic-based flexible packaging films.

Due to the maximum measurement capacity of the oxygen barrier device being 200 cm^3^/m^2^/day, films with oxygen permeability exceeding this limit are reported as having a permeability of 200 cm^3^/m^2^/day. Upon evaluating the oxygen barrier data and graphs, it has been observed that polyolefin-based films, such as PE and BOPP, commonly used in the current flexible packaging industry, lack significant oxygen barrier properties. In contrast, PET film exhibits an oxygen barrier with a permeability of 128.94 cm^3^/m^2^/day. Additionally, OPA film, another type of plastic-based film, demonstrates an oxygen barrier with a permeability of 5.40 cm^3^/m^2^/day. The aluminum film shows the lowest barrier value, measured at 0.25 cm^3^/m^2^/day. Given the limited barrier properties of plastic films, they are often utilized in flexible packaging structures by either laminating them with aluminum or coated films.

When examining biobased films, it was noted that PLA did not exhibit oxygen barrier properties comparable to those of conventional polyolefin films. However, the application of a gelatin coating significantly improved the barrier performance of PLA, reducing its oxygen permeability to 16.19 cm^3^/m^2^/day. As a result, gelatin-coated PLA films show promise as sustainable alternatives to traditional packaging materials such as PET, BOPP, and PE, offering enhanced protection against oxygen transmission.

Furthermore, applying a gelatin coating to films that already possess barrier functionality—such as cellulose, with an initial OTR of 2.20 cm^3^/m^2^/day further enhanced their barrier performance, reducing permeability to 0.64 cm^3^/m^2^/day. This improved barrier is comparable to that of aluminum films. These findings demonstrate that a gelatin coating can elevate the performance of existing barrier films to levels similar to nonrecyclable materials like aluminum, providing a viable and more sustainable alternative for packaging applications

### 3.2 Mechanical Test Results

To evaluate the mechanical strength of both coated and uncoated films, tensile strength tests were conducted using a Lloyd LS model device. The tensile and extension behaviors of the films were measured under applied stress. For this test, samples with a width of 25 mm and equal length were prepared.

According to the mechanical test data shown in Table 2, uncoated cellulose and PLA films exhibit distinct mechanical properties. Among them, cellulose film demonstrated the highest applied load at maximum load (56.37 N) and the highest stiffness value (32,113.0 N/m). PLA followed with a maximum load of 33.36 N and a stiffness value of 15,356.67 N/m. These results indicate that while PLA is less stiff and bears lower maximum load than cellulose, it still offers considerable mechanical strength.

**Table 2:** Tensile Strength Test Results of Coated and Uncoated PLA and Cellulose Films

parameters	coated PLA	PLA	coated cellulose	cellulose
load at maximum load (N)	39.84 ± 1.12	33.36 ± 0.98	66.27 ± 1.65	56.38 ± 1.44
extension at maximum load (mm)	2.64 ± 0.14	3.68 ± 0.22	6.13 ± 0.29	11.13 ± 0.38
stiffness(N/m)	21,608.67 ± 742.15	15,356.67 ± 645.78	34,788 ± 1220.52	32,113 ± 1165.44

Moreover, cellulose film displayed the highest extension at maximum load (11.13 mm), compared to PLA (3.68 mm). The fact that cellulose film, despite its high stiffness, also exhibited greater extension suggests that it retains a certain level of flexibility and deformation capacity. This dual behavior has been previously reported for cellulose-based films, which can exhibit both rigidity and ductility depending on moisture content and structural organization.^18^ Increased extension at maximum load is typically associated with improved elasticity and flexibility properties that can be advantageous in packaging applications where stretchability is desired.^12^

When gelatin was applied to these biobased films, both the load and stiffness at maximum load increased in PLA and cellulose films, while extension at maximum load decreased. This pattern suggests that gelatin reinforces the mechanical integrity of the films, enhancing their structural rigidity and reducing their capacity to stretch under applied force. Similar findings were reported by Li et al.,^12^ where gelatin coatings significantly improved the mechanical properties of biobased films by increasing tensile strength while reducing extension. Debeaufort et al. also observed that gelatin-based coatings improved the stiffness of PLA films through cross-linking effects, which restrict polymer chain mobility and enhance load-bearing capacity.^12^ The reduction in extension observed in the current study further supports this mechanism, as increased intermolecular interactions between gelatin and the film surface likely contribute to a more rigid structure. Overall, these results confirm that gelatin coatings can enhance the mechanical performance of biobased films by improving their load resistance and stiffness. However, the accompanying reduction in extension may influence the flexibility required for specific packaging applications.

To observe the effect of gelatin coating on the puncture strength of PLA and cellulose films, puncture strength tests were conducted comparatively from outside to inside and from inside to outside, both with and without coating. The results are presented in Table 3.

**Table 3:** Puncture Test Results of Coated and Uncoated PLA and Cellulose Films

	outside to inside		inside to outside	
film	load at break (N)	extension (mm)	load at break (N)	extension (mm)
**PLA**	14.70 ± 0.71	11.33 ± 0.45	15.56 ± 0.06	11.78 ± 1.00
**coated PLA**	18.17 ± 3.29	14.51 ± 0.64	13.88 ± 0.65	12.63 ± 0.23
**cellulose**	10.73 ± 0.41	11.03 ± 0.49	11.21 ± 0.35	10.41 ± 1.61
**coated cellulose**	13.19 ± 0.15	10.09 ± 0.50	11.51 ± 1.22	9.54 ± 0.47

While the puncture strength measured from outside to inside reflects the film’s resistance to forces applied to its outer surface, the puncture strength measured from inside to outside indicates its resistance to forces applied to the film’s inner surface. The gelatin coating was applied to the outer surface of the films. According to Table 4, when comparing PLA and cellulose films, it was found that the puncture strength of both films increased with the gelatin coating.

**Table 4:** Optical Test Results of Coated and Uncoated Films

film type	gloss	haze	clarity	transmittance
**PLA**	141 ± 0.82	2.60 ± 0.08	95.00 ± 0.26	93.20 ± 0.30
**coated PLA**	150 ± 1.00	3.02 ± 0.23	94.97 ± 0.21	92.03 ± 0.06
**cellulose**	158.67 ± 1.53	6.82 ± 0.19	93.57 ± 0.31	89.80 ± 0.26
**coated cellulose**	156 ± 1.00	4.75 ± 0.21	94.27 ± 0.32	90.13 ± 0.15

The increased load at break and stiffness observed in the tensile tests generally indicate enhanced material durability. Therefore, the puncture strength of the gelatin-coated PLA and cellulose films likely increased, as higher load at break and stiffness contribute to improved material strength, corresponding to puncture resistance.

It should also be noted that the gelatin coating provides flexibility and stickiness, which can affect load distribution on the film surface and potentially alter puncture strength. As a result, the effect of the gelatin coating on puncture strength varies depending on the type of polymer, coating thickness, and application method.

In measurements taken from inside to outside, different effects were observed between the two films. For these inside–outside measurements, it was found that the gelatin coating did not change the load at break of the cellulose film; however, the load at break decreased when applied to the PLA film. The extension at break measured from inside to outside increased in gelatin-coated PLA films but decreased in cellulose films. This indicates that inside–outside measurements differ from outside–inside measurements, likely due to variations in the formulations on the inner and outer sides of the films, which in turn affect their load at break.

### 3.3 Optical Test Results

As packaging is the first element to catch the eye, these optical features assist consumers in forming a positive perception of the product’s quality, freshness, and reliability. Optical properties such as gloss, haze, clarity, and transmittance play a crucial role in determining the quality of packaging films. To assess the impact of gelatin on the optical properties of the base film, measurements of gloss, haze, clarity, and transmittance were conducted on both coated and uncoated films. The results are presented in Table 4.

Analyzing the data presented in Table 4, it is evident that the gloss values of gelatin-coated films are higher than those of uncoated PLA and cellulose films, indicating that the gelatin coating enhances the gloss of the film surface. Furthermore, the observation that gelatin coating increases haze in PLA film while decreasing it in cellulose film suggests that the interaction between gelatin and these biodegradable polymers leads to varying optical properties on the film surface after coating.

Haze on the surface of plastic films arises from light scattering or reflection. The gelatin coating may increase haze in PLA films by inducing more scattering or reflection upon application, thereby introducing visual uncertainty. Conversely, the reduction in haze observed in cellulose films could stem from the gelatin coating forming a more homogeneous layer on the film surface, resulting in less light scattering or reflection. This discrepancy may be attributed to different interactions of gelatin with the polymers and the subsequent changes in the structural and optical properties of the film surface postcoating.

Regarding clarity and transmittance values, minimal differences are observed between coated and uncoated films, indicating similar clarity and light transmittance. However, it is important to note that the performance of each film in industrial applications may vary, particularly after the printing process.

Gelatin is a yellow material naturally. However, as the coating formulation is diluted with water, the gelatin becomes lighter and more transparent, which should not cause visible color differences in the printing industry. To observe the effect of color on the printed film, cyan and magenta from the CMYK color model were applied to two PLA films. A gelatin coating was applied to one of the cyan and magenta films, as shown in Figure 7.

**Figure 7:**
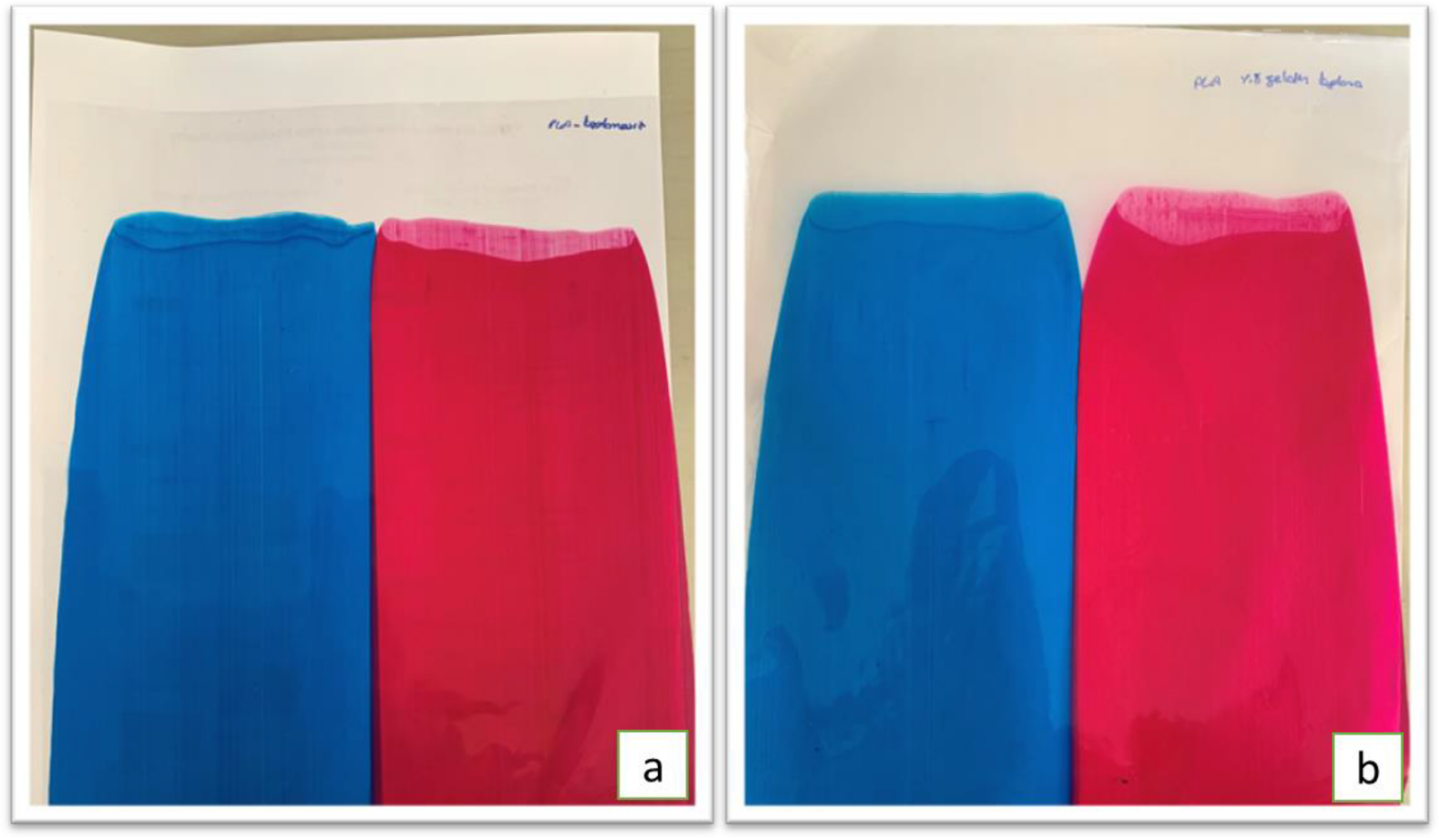
(a) Cyan and magenta ink applied PLA film and (b) gelatin applied over magenta and cyan ink.

In the printing process, *L*, *a*, *b*, and Δ*E* measurements play an important role in evaluating print quality and ensuring color consistency. According to the ISO 12647-2:2013 standard, the Δ*E* limit for acceptable color variation in the printing process is generally set at 5.^19^ A lower Δ*E* value indicates a smaller difference between colors, reflecting acceptable color consistency. The *L*, *a*, *b*, and Δ*E* measurement data used to observe the color difference after coating are presented in Table 5.

**Table 5:** Color Measurement Results of Printed Films before and after Gelatin Coating Application

	PLA		coated PLA	
	cyan	magenta	cyan	magenta
**L**	44.58 ± 0.70	42.65 ± 0.25	44.69 ± 0.83	41.71 ± 0.21
**a**	–30.64 ± 1,16	76.27 ± 0.30	–31.26 ± 1.22	76.61 ± 0.49
**b**	–62.60 ± 0.34	9.21 ± 0.41	–63.78 ± 0.41	11.24 ± 1.09
**Δ*E***			2.66	2.73

When one of the cyan and magenta applied PLA films was coated with gelatin, no significant difference was observed in the *L* and *a* values. Only a minor increase was noted in the *b* value of the magenta color, which changed from 9.21 without coating to 11.24 with coating. In the Δ*E* measurements conducted to assess the difference between coated and uncoated colors, a Δ*E* of 2.66 was found for cyan, while Δ*E* for magenta was measured at 2.73. Although the ISO 12647-2:2013 standard sets the Δ*E* limit at 5, the lower color differences indicate consistency between the colors, suggesting that the variation is quite small. Thus, the numerical data demonstrate that the color difference in the coated films is not distinguishable by the naked eye and is acceptable for the printing process.

## 4 Conclusions

This study aimed to develop a biobased coating material using gelatin to enhance the oxygen barrier and mechanical properties of biobased films, including PLA and cellulose. By varying the ratios of gelatin, glycerol, and Tween 80, the effects of different component concentrations on the barrier and mechanical properties of the coated films were evaluated. The optimal formulation, yielding the lowest oxygen permeability and standard deviation, was identified as 8% gelatin, 2% glycerol, and 0.1% Tween 80.

Results demonstrated that the gelatin coating significantly improved oxygen barrier properties, reducing permeability from over 200 to 16.19 cm^3^/m^2^/day for the PLA film, and from 2.20 to 0.64 cm^3^/m^2^/day for the cellulose film at 23 °C and 80% RH. Tensile tests revealed that the gelatin coating enhanced the durability and strength of the films. The increased force at maximum load indicated that the coating improved the packaging’s load-bearing capacity, providing greater resistance and increased hardness, which also enhanced surface scratch resistance.

Regarding optical properties, coated films were generally shinier, while clarity and permeability remained similar to uncoated films. The effect of the coating on print color was assessed, with Δ*E* values between 2.66 and 2.73, indicating that color differences in gelatin-coated films were not perceptible, as Δ*E* remained below the ISO 12647-2:2013 threshold of 5.

Overall, this study highlights the potential of biobased gelatin coatings to improve the sustainability and performance of flexible packaging materials, supporting the adoption of environmentally friendly practices in the packaging industry.
